# Structure-dependent growth control in nanowire synthesis via on-film formation of nanowires

**DOI:** 10.1186/1556-276X-6-196

**Published:** 2011-03-04

**Authors:** Wooyoung Shim, Jinhee Ham, Jin-Seo Noh, Wooyoung Lee

**Affiliations:** 1 Department of Materials Science and Engineering, Yonsei University, 134 Shinchon, Seoul 120-749, Korea; 2 Department of Materials Science and Engineering, Northwestern University, Evanston, IL 60208-3108, USA

## Abstract

On-film formation of nanowires, termed OFF-ON, is a novel synthetic approach that produces high-quality, single-crystalline nanowires of interest. This versatile method utilizes stress-induced atomic mass flow along grain boundaries in the polycrystalline film to form nanowires. Consequently, controlling the magnitude of the stress induced in the films and the microstructure of the films is important in OFF-ON. In this study, we investigated various experimental growth parameters such as deposition rate, deposition area, and substrate structure which modulate the microstructure and the magnitude of stress in the films, and thus significantly affect the nanowire density. We found that Bi nanowire growth is favored in thermodynamically unstable films that facilitate atomic mass flow during annealing. A large film area and a large thermal expansion coefficient mismatch between the film and the substrate were found to be critical for inducing large compressive stress in a film, which promotes Bi nanowire growth. The OFF-ON method can be routinely used to grow nanowires from a variety of materials by tuning the material-dependent growth parameters.

## Introduction

Recently, we reported a new nanowire growth method, termed on-film formation of nanowires (OFF-ON), that combines the advantages of simple thin film deposition and whisker formation to achieve highly crystalline nanowires [1]. OFF-ON is a template- and catalyst-free synthetic approach that utilizes thermally induced compressive stress within a polycrystalline thin film to obtain nanowires as small as tens of nanometers in diameter. Because of its direct growth capability via atomic mass flow and compatibility with multi-component materials, OFF-ON can be used to grow, sequentially or in parallel, single-element [1] and multiple compound nanowires [2]. Importantly, there is no need to use catalysts, thus avoiding cross-contamination that degrades the properties of the resultant nanowires. These capabilities make OFF-ON a unique and highly desirable tool for growing defect-free, high-quality and single-crystalline nanowires composed of a material of interest.

The first demonstration of OFF-ON was carried out with bismuth (Bi) nanowires [1]. Unlike other methods [3-10], typical Bi nanowires grown by OFF-ON are as long as hundreds of micrometers with exceptional uniformity in diameter and can be used as unique building blocks linking integrated structures over large length scales. The advantage of using OFF-ON to grow Bi nanowires has been demonstrated by oscillatory and nonoscillatory magnetoresistance measurements that show that nanowires grown via OFF-ON are high-quality single-crystalline [11,12]. Subsequently, OFF-ON has been expanded to grow a wide variety of materials and structures, including Bi_2_Te_3 _[2], Bi-Te core/shell [Kang J, Roh JW, Ham J, Noh J, Lee W: Reduction of thermal conductivity in single Bi-Te core/shell nanowires with rough interface, submitted], Bi-Te superlattice [Kang J, Ham J, Noh J, Lee W: One-dimensional structure transformation by on-film formation of nanowires: Bi-Te core/shell nanowires to Bi/Bi_14_Te_6 _multi-block heterostructure, submitted], nanoparticle-embedded [Ham J, Roh J, Shim W, Noh J, Lee W: Nanostructured Thermoelectric Materials: Al_2_O_3 _nanopartice-embedded Bi Nanowires for ultra-low thermal conductivity, submitted], and self-assembled Bi nanowires [13]. OFF-ON is a promising nanowire growth platform; however, factors that ultimately control many important growth parameters to increase nanowire density have not been investigated. Herein, the authors report the effect of various parameters on Bi nanowire growth. The parameters studied were the microstructure and size of the as-deposited Bi films and the substrate structures on which they were deposited. Clarification of such effects provides optimized conditions for achieving high nanowire densities for specific applications.

## Experimental details

**Figure 1:**
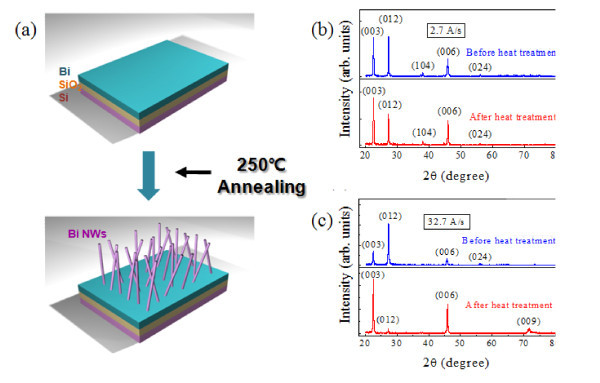
**Growth and X-ray diffraction (XRD) patterns of Bi sputtered films**. **(a)** Schematic representation of the growth of Bi nanowires by OFF-ON. XRD patterns of Bi films before and after heat treatment at 270°C for 10 h. The films were deposited at a rate of **(b)** 2.7 Å/sec (RF power: 10 W) and **(c)** 32.7 Å/s (RF power: 100 W), respectively.

Bi nanowires were fabricated by the OFF-ON method simply by annealing a Bi film at relevant temperatures without the use of conventional templates, catalysts, or starting materials (Figure 1a). Details related to the preparation of the substrates, deposition of the thin films, and annealing procedure are presented in [1]. In this study, the effect of several major parameters on Bi nanowire growth was examined. First, the effect of the Bi film microstructure, which can be modulated by film deposition rate, on the growth of nanowires was investigated. For this purpose, Bi thin films were deposited onto thermally oxidized Si (100) substrates at deposition rates of 2.7 Å/s (RF power: 10 W) and 32.7 Å/s (100 W), using UHV radio frequency (RF) sputtering. Second, the effect of Bi film areas, where the Bi nanowires are grown, on nanowire density was addressed. To study this, Bi films of various areas were fabricated using photolithography and lift-off. Four different Bi film areas were tested: (10^4 ^μm)^2^, (10^3 ^μm)^2^, (10^2 ^μm)^2^, and (10 μm)^2^. Third, we examined the effect of the magnitude of the compressive stress on the Bi film, which is modulated by the thermal expansion of the substrate, on Bi nanowire density. For this study, two different substrates, i.e., a thermally oxidized Si substrate and a Si substrate without SiO_2 _on top were used.

Bi nanowires and Bi thin films were characterized by high-resolution X-ray diffractometer (Rigaku D/MAX-RINT, XRD), atomic force microscopy (DI 3100 AFM with a Nanoscope IVa controller), scanning electron microscopy (FE-SEM JEOL 6701F), and optical microscope (Olympus OM). Topology of Bi thin films deposited at rates of 2.7 and 32.7 Å/s were examined by contact-mode AFM after heat treatment. To calculate the Bi nanowire density, each Bi thin film was divided into 16 parts. Then, the number of nanowires on two randomly selected parts was counted using OM, and the average nanowire density was calculated.

## Results and discussion

**Figure 2:**
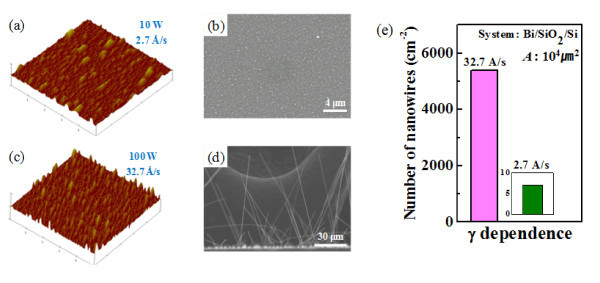
**AFM images (5 μm × 5 μm in size) of Bi films **deposited at a rate of **(a)** 2.7 Å/s and** (c) **32.7 Å/s, after heat treatment at 270°C for 10 h, **(b, d)** SEM images of the respective Bi films, with no nanowires and dense nanowires on them, **(e)** Histograms of Bi nanowire densities depending on the deposition rates.

Figure 1b,c show X-ray diffraction (XRD) patterns of Bi thin films grown at deposition rates (γ) of 2.7 Å/s (RF power: 10 W) and 32.7 Å/s (RF power: 100 W), respectively, before and after thermal annealing. For both deposition rates, the identical 50-nm-thick Bi films were obtained by controlling the deposition time. From Figure 1b,c, it is evident that the Bi film grown at 100 W have preferential orientations of (003), (006), and (009) after heat treatment, while the film deposited at 10 W have additional orientations of (012) and (104). Interestingly, Bi nanowires grew from Bi films deposited at 100 W at far higher densities than from Bi films deposited at 10 W (see Figure 2). This implies that the preferential orientation (00ℓ) in a Bi film facilitates Bi nanowire growth. At a fixed growth temperature, the impinging flux of Bi atoms onto the surface of a substrate is expected to be higher for the higher RF power of 100 W, leading to a shorter time interval between encounters of adatoms, and in turn, creating a local excess of adatoms, called supersaturation [14]. This causes adatoms not to settle into possible equilibrium positions, resulting in the Bi film having a non-equilibrium microstructure and a non-uniform surface. In such a Bi film, Bi atoms are more likely to occupy unstable positions and are susceptible to migration upon thermal activation. This is why the grain orientations of the Bi film deposited at 100 W are redirected to the (00ℓ) through thermal annealing, as shown in Figure 1c.

The inference above is more directly observed from the AFM images. Figure 2a,b shows AFM images of annealed Bi thin films grown at rates of 2.7 Å/s (10 W) and 32.7 Å/s (100 W). The film grown at 100 W is rougher and shows a greater number of protrusions on the surface compared to the film deposited at 10 W. Figure 2c,d shows SEM images of Bi nanowires grown on annealed Bi thin films that were initially deposited at rates of 2.7 and 32.7 Å/s, respectively. In contrast with the case of the film grown at 2.7 Å/s where few nanowires are observed, many long Bi nanowires are found on the Bi film deposited at 32.7 Å/s. Figure 2e shows that the ratio of the Bi nanowire densities for the two cases reaches approximately 800. Based on a localized model [15], the surface oxide layer may strongly affect nanowire growth because a nanowire can grow only when it can break the naturally formed oxide layer at the cost of stored compressive stress. The surface oxide layer is less likely to form on sharp protrusions. Therefore, we assume that a higher density of Bi nanowires can be achieved on films grown at a higher deposition rate partly because of Bi films with a higher density of protruding regions that can easily break the surface oxide layer at a given compressive stress. Moreover, a high deposition rate tends to induce a fine grain structure because of the limited surface migration of adatoms as mentioned before, and Bi atomic diffusion during thermal annealing is expected to be favored for nanowire growth through enlarged grain boundaries. These results indicate that surface morphology and grain structure of the Bi film, along with the preferential orientations stated in Figure 1, are critical factors in determining how easily Bi nanowires can grow on it. Consequently, the deposition rate of a Bi film is a parameter of importance, which controls all of these factors; a high deposition rate promotes Bi nanowire growth.

**Figure 3:**
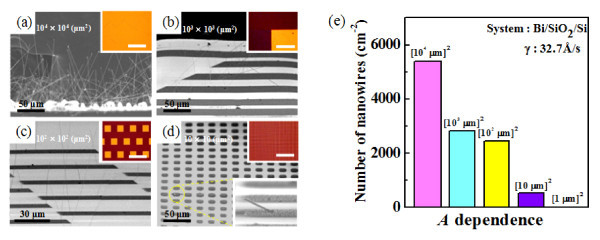
**SEM images of Bi nanowires grown on Bi films with different areas**: **(a)** (10^4 ^μm)^2^, **(b)** (10^3 ^μm)^2^, **(c) **(10^2 ^μm)^2^, and **(d)** (10 μm)^2^. Insets show optical microscope images of the samples before annealing. (e) Histograms of Bi nanowire densities depending on the Bi film areas.

Compressive stress stored in Bi films is thought to be the driving force for spontaneous Bi nanowire growth by the OFF-ON method. In order to check the appropriateness of this hypothesis and to study the effect of another parameter on Bi nanowire growth, we investigated the effect of Bi film areas. For this, we fabricated Bi thin film patterns with four different size of areas: (10^4 ^μm)^2^, (10^3 ^μm)^2^, (10^2 ^μm)^2^, and (10 μm)^2^. Figure 3a,b,c,d shows SEM images of Bi nanowire grown on different Bi film areas (*A*), where the Bi films were deposited on SiO_2_/Si substrates at a rate of 32.7 Å/s. If the compressive stress hypothesis is reasonable, then a larger Bi film area should result in a higher density of Bi nanowires, because the compressive stress is generally less relieved at the center of a film and more released at the edges of the film. Indeed, we found that the density of Bi nanowires at the edge is higher in the factor of 1.3 than that at the center, and the total density increased as the Bi film area increased after annealing at 270°C for 10 h (see Figure 3e). This indirectly shows that compressive stress is a driving force for Bi nanowire growth by the OFF-ON method, and preventing stress relief is another key factor for promoting nanowire growth. In this sense, Bi film area is another parameter that determines the Bi nanowire density. The magnitude of stress and its correlation with the nanowire density is discussed in detail elsewhere [16]. In addition, the above result proves that Bi nanowire growth is not driven by the thermal evaporation of Bi atoms during annealing; if this were the case, then Bi nanowire density should be independent of Bi film area.

**Figure 4:**
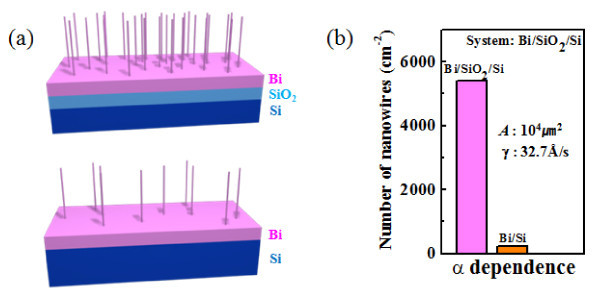
**Schematics and histograms of Bi nanowire densities**. **(a)** Schematics of Bi nanowires grown on different substrates. **(b)** Histograms of Bi nanowire densities depending on the substrate structures.

Finally, the effect of the substrate layer structure (α) on Bi nanowire density was investigated to elucidate the role of thermal expansion mismatch between the substrate and the film. For this study, two different film stack structures, Bi/SiO_2_/Si and Bi/Si, with different thermal expansion mismatches, were exploited. Here, Bi films were deposited at an identical rate of 32.7 Å/s for both stacks. Figure 4a schematically shows Bi nanowires grown on the Bi/SiO_2_/Si and Bi/Si stacks, illustrating that the nanowire density on a Bi/SiO_2_/Si stack is much larger than on a Bi/Si stack. In fact, the Bi nanowire density on the Bi/SiO_2_/Si stack was measured to be 5400 cm^-2^, which is much higher than that on the Bi/Si stack (240 cm^-2^), as shown in Figure 4b. The thermal expansion mismatch that causes compressive stress in a film results from the large difference in thermal expansion coefficients of Bi (13.4 × 10^-6^/°C) and SiO_2 _(0.5 × 10^-6^/°C) or Si (2.4 × 10^-6^/°C). It is inferred that the 20 times larger Bi nanowire density on the Bi/SiO_2_/Si stack results from the larger mismatch of thermal expansion coefficients between the substrate and the Bi film for the Bi/SiO_2_/Si stack than for the Bi/Si stack (note the difference in the thermal expansion coefficients of Si and SiO_2_). Therefore, the choice of a substrate structure that can maximize the thermal expansion mismatch with the film is a crucial parameter for optimizing nanowire growth. This principle may be universally applicable to nanowire growth based on any material systems, using the OFF-ON method.

## Conclusions

We have investigated the effect of major growth parameters on Bi nanowire growth by the OFF-ON method. It was found that a rough Bi film surface and a fine Bi film grain structure induced by a high deposition rate facilitate Bi nanowire growth. The Bi nanowire density increases as the size of Bi film area increases and as the difference in thermal expansion coefficients between the substrate and the Bi film increases, confirming that compressive stress acts as the driving force for Bi nanowire growth by the OFF-ON method. These results indicated that major parameters should be properly set to achieve the highest density of Bi nanowires, using the OFF-ON. The OFF-ON method can be used equally well for growth of nanowires from other materials by adjusting these major growth parameters.

## Abbreviations

Bi: bismuth; RF: radio frequency; XRD: X-ray diffraction.

## Competing interests

The authors declare that they have no competing interests.

## Authors' contributions

The work presented here was carried out in collaboration between all authors. WS, JH and WL defined the research theme. WS and JH designed methods and experiments, carried out the laboratory experiments, analyzed the data, interpreted the results and wrote the paper. J-SN co-worked on associated data collection, their interpretation and wrote the paper. WL co-designed experiments, discussed analyses, and wrote the paper. All authors have contributed to, seen and approved the manuscript.
